# Immunotherapy with CAR-Modified T Cells: Toxicities and Overcoming Strategies

**DOI:** 10.1155/2018/2386187

**Published:** 2018-04-17

**Authors:** Shangjun Sun, He Hao, Ge Yang, Yi Zhang, Yang Fu

**Affiliations:** ^1^Department of Gastroenterology, The First Affiliated Hospital of Zhengzhou University, Zhengzhou, Henan 450052, China; ^2^Department of Oncology, Ansteel Group Hospital, Anshan, Liaoning 114000, China; ^3^Department of Orthopedics, Ansteel Group Hospital, Anshan, Liaoning 114000, China; ^4^Department of Ophthalmology, The First Affiliated Hospital of Zhengzhou University, Zhengzhou, Henan 450052, China; ^5^Biotherapy Center, The First Affiliated Hospital of Zhengzhou University, Zhengzhou, Henan 450052, China

## Abstract

T cells modified via chimeric antigen receptors (CARs) have emerged as a promising treatment modality. Unparalleled clinical efficacy recently demonstrated in refractory B-cell malignancy has brought this new form of adoptive immunotherapy to the center stage. Nonetheless, its current success has also highlighted its potential treatment-related toxicities. The adverse events observed in the clinical trials are described in this review, after which, some innovative strategies developed to overcome these unwanted toxicities are outlined, including suicide genes, targeted activation, and other novel strategies.

## 1. Introduction

Cell-based therapies have risen to the forefront of treatment approaches for cancer [1]. Progress in synthetic biology and gene transfer enables a rapid and efficient redirection of polyclonal T lymphocytes [2]. T cells modified via synthetic CARs have made remarkable achievements in eliminating chemotherapy-resistant acute lymphoblastic leukemia [3–7], chronic lymphocytic leukemia [8, 9], and non-Hodgkin lymphoma [10, 11]. In light of their promise, there has formed a broad wave of CAR-modified T cells for cancer immunotherapy, including the challenging solid tumors [12–15].

CARs commonly composed of an extracellular antigen-binding moiety (i.e., single-chain variable fragment of antibody) fused to intracellular signaling domains can reprogram specificity against the targeted molecules of a selected cell and outsmart HLA restriction [16, 17]. Upon antigen ligand engagement, CAR T cells can produce cytokines, kill targeted cells, and stimulate the proliferation of T cells, resulting in a highly amplified response and the consequent eradication of a huge quantity of tumor cells within weeks. Despite CAR T cells being promising, toxicities have been associated with most of the clinical responses, and fatal complications have been observed in some patients treated with gene-modified T cells [18–22]. The aim of this review is to provide a framework for the classification of different toxicities and highlight state-of-the-art potential overcoming strategies.

## 2. Toxicities of T Cells Genetically Modified with CARs

**Figure 1:**
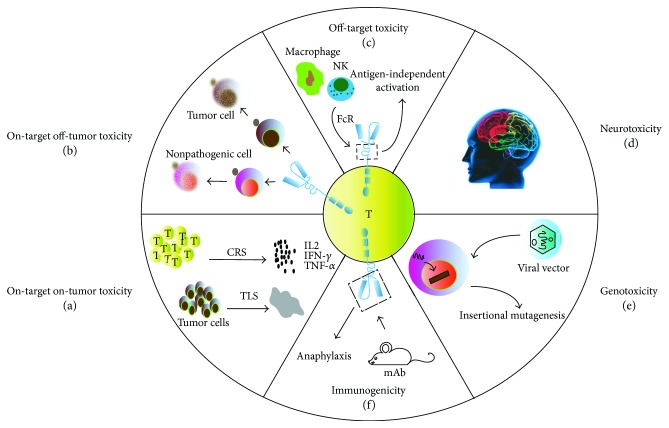
Toxicities of T cells genetically modified with CARs. (a) On-target on-tumor toxicity. (a1) Effector T-cell activation and excessive cytokine release may result in cytokine release syndrome (CRS). (a2) High tumor load leads to massive destruction of tumor tissue, resulting in tumor lysis syndrome (TLS). (b) On-target off-tumor toxicity: the shared target antigen is also expressed on nonpathogenic cell, subsequently damaging healthy tissue. (c) Off-target toxicity: the extracellular crystallizable fragment (Fc) of CARs can interact with the Fc receptor (FcR) expressed on innate immune cells, leading to antigen-independent activation. (d) Neurotoxicity: manifestation ranges from confusion, delirium, aphasia to some degree of myoclonus, and seizure. (e) Genotoxicity: integrating viral vectors used to facilitate the stable expression in primary T cells may pose a potential risk of oncogenic insertional mutagenesis. (f) Immunogenicity: single-chain variable fragments (scFvs) derive from mouse monoclonal antibodies (mAbs), leading to severe immune response.

A brisk immune response can be a double-edged weapon. The efficacy of T cells genetically modified with CARs against cancer is greatly improved at the expense of enhanced toxicities; therefore, it will be useful to classify the multifaceted adverse events in trials, clearly dividing them into five categories, i.e., on-target on-tumor, on-target off-tumor, off-target, neurotoxicity, and other toxicities (Figure 1).

### 2.1. On-Target On-Tumor Toxicity

When it comes to the toxicity specific to the administration of T cells itself, the most common toxicity is the on-target on-tumor type, which is triggered by excessive cytokine release or tumor cell necrosis (Figure 1(a)). The underlying premise of immunotherapy is to activate effector T cell and achieve cytokine release. However, excessive cytokine release may result in cytokine release syndrome (CRS), which can vary from mild moderate to severe potentially fatal forms [18–20]. Furthermore, the rapid devastation of large quantities of tumor cells can also trigger tumor lysis syndrome (TLS), which can bring out an array of systemic metabolic disturbances with an overlap in symptoms with CRS and is characterized by elevated levels of phosphate, potassium, and uric acid in serum [8, 21]. Emerging evidence suggests that the severity of CRS and TLS depends upon disease burden [3, 22]; splitting the initial dose and strictly monitoring the vital parameters can mitigate the risk [5, 23]. Additionally, considering that CRS manifests as a rapid immune reaction driven by the massive release of cytokines, including IFN-*γ*, IL-6, and IL-10, the administration of high-dose corticosteroids and corresponding antagonist mAb (e.g., IL-6 receptor antagonist mAb and tocilizumab) can also be effective therapeutic interventions [24–26].

### 2.2. On-Target Off-Tumor Toxicity

The most striking toxicity specific to genetically targeted T cells is “on-target off-tumor,” resulting from a direct attack on normal tissues that have the shared expression of the targeted antigen (Figure 1(b)). Considering the potency of redirected T cells, toxicity on nonpathogenic tissues expressing low levels of the antigen can be highly detrimental. For example, Erasmus University's earliest trials described the occurrence of cholestasis in renal cell carcinoma patients infused with T cells modified with a CAR specific for carbonic anhydrase IX, which is physiologically expressed on bile duct epithelial cells [27, 28]. Similarly, low-level ERBB2 expression on lung epithelia might have precipitated the reported case of fatal lung toxicity [29]. With these toxicities in mind, the selection of target antigen, which is strictly specific to the tumor (e.g., EphA2 [30] and mutated EGFRvIII [31, 32]) or on the category of nonessential tissues (e.g., thymic stromal lymphopoietin [33, 34] and CD33 [35, 36]), is probably the most critical determinant to broaden the application. Indeed, such antigens have been difficult to identify, particularly in the settings of solid malignancies. Moreover, a study proved that the substantial dose of infused CAR T cells (1 × 10^10^) could potentially provoke this toxicity, and lower doses of HER2/neu-specific CAR T cells (without prior conditioning chemotherapy) were safe [13]. Hence, given the known background expression of the target antigen, it becomes extremely important to determine whether levels are over the threshold that can cause this toxicity and to determine the potential severity thereof in humans.

### 2.3. Off-Target Toxicity

Off-target toxicity occurs when the transduced T-cell population unexpectedly attacks an antigen other than the intended one or activates themselves independently from their specificity (Figure 1(c)). The majority of CAR T cells recognize antigens through single-chain variable fragments derived from monoclonal antibodies (mAbs). However, the safety of some mAbs profile is uncertain. The data *in vitro* suggested that the artificial synthetic constructs themselves may carry some risks of off-target recognition. For example, the toxicity profile of the mAbs has been illustrated in the case of trastuzumab (anti-HER2/neu), in which CARs carrying the IgG1-derived CH2CH3 domain as extracellular spacer may interact with the Fc receptor expressed on innate immune cells (e.g., macrophages and NK cells), leading to antigen-independent activation [29]. Fortunately, the off-target recognition of cross-reactive antigens has not been evident in CAR T-cell trials to date. Nonetheless, fatal cardiac toxicity has been seen in 2/2 patients infused with autologous T cells engineered to express an enhanced affinity T-cell receptor (TCR) directed against the testis antigen MAGE-A3 [37, 38], of which the cross-reactivity occurred against titin only expressing in cardiac tissue [39]. Therefore, this possibility has to be kept in mind for future developments when CAR T cells target novel tumor-associated antigen.

### 2.4. Neurotoxicity

Neurotoxicity is another potentially serious toxicity observed in patients receiving CD19-specific CAR T-cell therapy, and its manifestation ranges from confusion, delirium, and aphasia to some degree of myoclonus and seizure (Figure 1(d)). What is not clear is the causative pathophysiology of these neurologic side effects. Although a clear expression of CD19 in the affected brain areas has not been shown, some groups have documented the infiltration of CAR T cells into the cerebrospinal fluid (CSF) in most patients with neurotoxicity [3–5, 40]. Lee et al. particularly found that 6/21 patients who had neurotoxicity had higher concentrations of CSF CAR T cells. However, magnetic resonance imaging scans often did not show abnormalities. Furthermore, a similar constellation of symptoms has also been observed in patients treated with blinatumomab [41, 42]. Therefore, it is uncertain if the toxicity arises from direct CAR T cells attack on the CNS tissue or generalized cytokine-mediated inflammation [43]. To date, the neurologic toxicity in all but the rare fatal cases has been reversible and self-limited. Understanding the mechanisms behind it will be critical for safer CAR T-cell therapy as well as for more effective management of these adverse effects.

### 2.5. Other Toxicities

Besides the toxicities mentioned above, there are some others as follows: (1) Immunosuppression: Immunosuppressive pretreating to the recipients prior to T-cell infusion is associated with much greater antitumor efficacy [44]. Unfortunately, the lymphodepleting and nonmyeloablative regimen comes along with the well-known toxicities of anemia, coagulopathy, and neutropenic sepsis. The mortality of this toxicity is approximately 1% and constitutes the major fatal risk of adoptive T-cell therapy in the National Cancer Institute Surgery Branch experience [45, 46]. (2) Immunogenicity: The majority of the antigen recognition region used in a genetically modified T cell is derived from mouse mAb [47], of which the foreign potential immunogenicity may lead to severe anaphylaxis [48–50] (Figure 1(f)). The mesothelin-specific CAR T cell had been reported to cause severe cardiac dysfunction [51], which was ultimately attributed to the formation of anti-mouse antibody triggered by allergic reaction. Therefore, diligent surveillance, prompt recognition, and immediate treatment must be adhered to, to control this life-threatening toxicity, whenever possible, and especially, if repeated dosing is planned, humanizing scFvs rather than mouse mAbs should be used [52]. (3) Genotoxicity: Integrating viral vectors used to facilitate the stable expression in primary T cells may pose a potential risk of oncogenic insertional mutagenesis, including the disruption of normal gene expression as observed in the therapy for SCID-X1 on the account of an uncontrollable LMO2 gene [53, 54] (Figure 1(e)). Though no such toxicity of vector-induced immortalization, clonal expansion, or enrichment for integration sites has been reported in CAR therapy to date [55], it is clearly an important consideration for the future when CAR T cells may prevail for the lifetime of the treated patient.

## 3. Overcoming Strategies of Related Toxicities

In light of the different spectrums of toxicities associated with the administration of T cells, it is logical to find a fine balance between tumor elimination and unexpected toxicities. To achieve this, innovative strategies have been implemented to offer compelling opportunities, including suicide gene, targeted activation, and other innovative gene therapy strategies.

### 3.1. Suicide Gene Therapy

**Figure 2:**
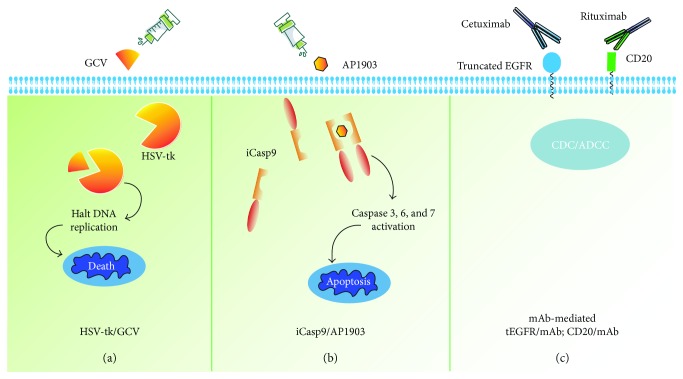
Summary of overcoming toxicities by the suicide gene co-expression in T cells. (a) HSV-tk turns the nontoxic prodrug GCV into GCV-triphosphate, leading to cell death by halting DNA replication. (b) iCasp9 can bind to the small molecule AP1903 and result in dimerization, which activates the intrinsic apoptotic pathway. (c) Targetable surface antigen expressed in the transduced T cells (e.g., CD20 and truncated EGFR), allowing eliminating the modified cells efficiently through complement/antibody-dependent cellular cytotoxicity (CDC/ADCC) after administration of the associated monoclonal antibody.

To manage unexpected toxicities or to eliminate transduced T cells after an eradication of the disease, coexpressing a conditional safety switch is a potentially effective tool. A suicide gene is a gene-encoding molecule, which allows the selective destruction of expressing cells upon the administration of a nontoxic prodrug and the elimination of the symptoms of treatment-driven toxicities (Figure 2); however, the clinical impact on their activation is unknown at present. The HSV-tk suicide gene has been utilized in most clinical settings, rendering target cells susceptible to GCV-mediated elimination [56–58] (Figure 2(a)). While limited by the immunogenicity of viral enzymes and the long time (several days) to reach full effect [59, 60], it may not be acceptable in the face of toxicities, which pose immediate threat to live. Alternative safety switches are based on the well-characterized, targetable surface antigen expressed in the transduced T cells, such as CD20 [61, 62] and truncated EGFR [63, 64], allowing eliminating the modified cells efficiently through complement/antibody-dependent cellular cytotoxicity (CDC/ADCC) after the administration of the associated monoclonal antibody (Figure 2(c)). Despite the preponderance of the system in nonimmunogenicity and dual-purpose nature of the additional transgene (which can also be used to measure the persistence of the transduced T cells), its efficacy and kinetics as a CAR T-cell elimination system have not been tested in the settings of clinical toxicities.

The inducible caspase 9 (iCasp9)/AP1903 suicide system is perhaps the most advanced and effective solution, which is based on the fusion of caspase 9 and a drug-sensitive FK-modified binding protein [60, 65]. Upon being exposed to the synthetic molecule AP1903, the fusion protein dimerizes and leads to the rapid apoptosis of T cells (Figure 2(b)). The efficacy and safety of iCasp9/AP1903 have been first demonstrated in allogeneic hematopoietic stem cell transplantation studies [66, 67]. When the GvHD occurred, AP1903 administration could eliminate iCasp9-expressing T cells within 30 min from the end of AP1903 administration (2 hours of infusion), followed by the permanent abrogation of symptoms without recurrence [68]. This response has also been replicated in preclinical models using CAR T cells along with coexpressing iCasp9 [69–71]. However, this represents the least preferred strategy, since the depletion of the CAR T cell will also mean abrogating its therapeutic potential, and the modulated activation of the switch and multiple administration of CAR T cells are potential strategies to overcome this issue.

### 3.2. Targeted Activation

#### 3.2.1. Targeting Two Tumor-Associated Antigens

**Figure 3:**
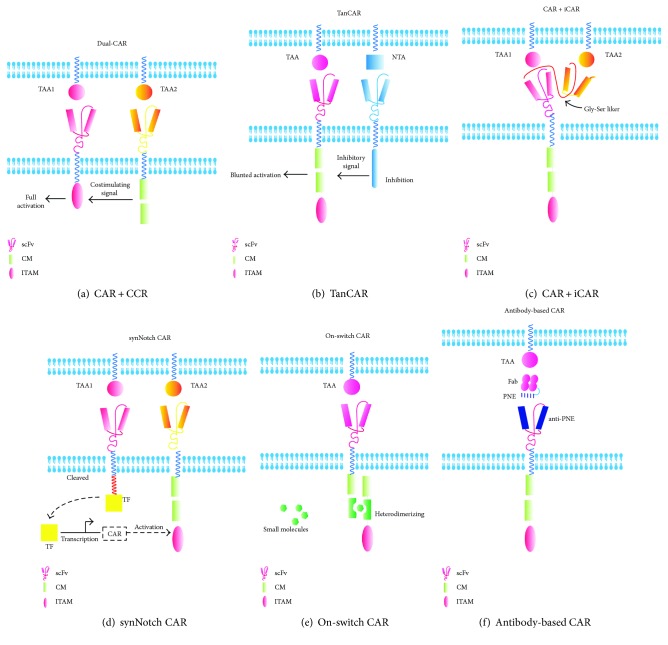
Summary of the targeted activation strategies for T cells to overcome toxicities. (a) In the dual targeting CAR-modified T cells, the T cells are transduced with both a CAR that provides suboptimal activation upon binding of one antigen and a chimeric costimulatory receptor (CCR) that recognizes a second antigen. (b) T cells are designed with a bispecific tandem CAR (TanCAR), in which two distinct antigen recognition domains are present in tandem by a Gly-Ser linker. (c) T cells can be engineered with an inhibitory receptor, carrying an intracellular domain from PD1 or CTLA-4, which can be triggered by an antigen expressed on normal cells, allowing T-cell inhibition outside the tumor. (d) Design of a synNotch AND-gate circuit that requires T cells to sense two antigens to activate. It works in two sequential steps: (1) The synNotch receptor is engineered to allow the T cell to recognize TAA1. Upon ligand recognition by the synNotch receptor, an orthogonal transcription factor is cleaved from the cytoplasmic tail, and (2) the T cell expresses a CAR directed towards TAA2. The cleaved transcription factor primes CAR expression. If A and B are present, the T cells can activate and kill the target tumor. (e) The on-switch CAR design distributes key components from the conventional CAR into two physically separate polypeptides that can be conditionally reassembled when a heterodimerizing small-molecule agent is present. Only in the presence of a heterodimerizing small molecule can they conditionally reassemble. (f) Antibody-based switches are engineered by the introduction of peptide neo-epitopes (PNE) at defined locations in an antigen-specific antibody. Given that the PNE is not an endogenous tissue or antigen , the activation of the sCAR-T cell is therefore strictly dependent on the presence of the switch. scFV: single-chain variable fragment of antibody; CM: costimulatory molecule; ITAM: immune-receptor tyrosine-based activation motif; TAA: tumor-associated antigen; NTA: normal tissue antigen; CAR: chimeric antigen receptor; CCR: chimeric costimulatory receptor; iCAR: inhibitory CAR; TanCAR: tandem CAR; synNotch CAR: synthetic notch CAR; TF: transcription factor; PNE: peptide neo-epitope; Fab: fragment of antigen binding.

Considering the prematurely attenuated therapeutic potential of suicide genes, there is a considerable interest in developing T cells whose activation can be controlled through combinatorial antigen-targeting activation with separated signals. These include dual targeting CAR strategies in which T cells are modified to express two CARs with different tumor-associated antigens to ensure that their activation occurs only on tumor cells [72–74]. It is achieved by “splitting” the activation signal and the endocostimulatory signal in different CAR constructs (Figure 3(a)). Likewise, this has also been proven in principle for Tan-CARs [75, 76], a single CAR that has specificity for two antigens owing to the expression of two tandemly arranged scFvs coupled to the same signaling domain (Figure 3(b)). Alternatively, if the presentation of antigens is exclusive to normal tissue, the inclusion of inhibitory CARs (iCARs) mediated by the physiological checkpoint molecule (PD-1 and CTLA-4) is another approach [77, 78]. The binding of iCARs bind to antigens found on normal cells can result in the inhibition of the CAR T-cell function, allowing a dynamic, self-regulating switch to target malignant tissue (expressing one antigen) while the normal tissue is spared [79] (Figure 3(c)). Recently, an novel dual-receptor AND-gate CAR called synthetic Notch (synNotch) has been developed in the lab of Wendell Lima, which consists of an engineered antigen-recognition domain towards an antigen of interest (e.g., CD19 or surface GFP), a Notch core, and an artificial transcription factor [80, 81]. Upon ligand recognition by the synNotch receptor, an orthogonal transcription factor (e.g., TetR-VP64 or Gal4-VP64) is cleaved from the cytoplasmic tail that regulates a custom genetic circuit, and the cleaved transcription factor primes CAR expression. Only when both antigens are present can it work orthogonally and requires no signaling intermediates, providing an extraordinary flexible way to regulate customized cascades in a wide variety of applications [82] (Figure 3(d)). However, the immunogenicity of the nonhuman transcription factors remains to be investigated [83].

#### 3.2.2. Switch-Mediated Activation

*(1) On-Switch CAR*. Overriding strategies by the inclusion of an “on-off” switch in CAR design enable the precise regulation of the location, duration, and intensity of therapeutic activities. Wu et al. [84] described an approach that gated cellular functions by clinician-prescribed small molecule inputs, making a major step. The authors distributed the conventional CAR into two parts by expressing the extracellular antigen-binding domain separately from the intracellular signal-transducing domain. Only in the presence of a heterodimerizing small molecule can they conditionally reassemble (Figure 3(e)). This approach has great potential for clinical application. Similarly, Juillerat et al. described a strategy to create a “transient” CAR T cells with a new architecture in CARs that are directly dimerized at the hinge domain with the addition of a small molecule. They finally confirmed that it can offer a basic framework to use alternative split-CARs and show a more controlled and potentially safer way towards the development of the engineered CAR T cell [85]. In summary, both exogenous control behaviors based on small molecules below can be implemented for the modified T cell to alter conventional T cells into smart T cells whose therapeutic behaviors are precise and effective and subject to user control [86].

*(2) Recombinant Antibodies as Switches*. With the rapid development of the bispecific antibodies in cancer immunotherapy [87, 88], the titratable recombinant antibody-based switches also enable the precise control geometry and stoichiometry of complex formation between the target cells and T cells. Examples of these switches include TAA-specific monoclonal antibodies that elicit antitumor activity from Fc-specific CAR T cells [89] and chemically or enzymatically modified antibody-hapten conjugates that redirect antihapten CAR T cells [90, 91]. Rodgers et al. reported the tumor antigen-specific Fab molecule engrafted with a peptide neo-epitope (PNE) that is bound exclusively by a PNE-specific switchable CAR T cell [92] (Figure 3(f)), and Kim et al. demonstrated the redirection of anti-FITC CAR T cells with a heterobifunctional small-molecule switch, folate-FITC, which selectively targets folate receptor-overexpressing cancers [93]. Overall, these switchable CAR T-cell dosing regimens could be tuned to provide efficacy comparable to that of the corresponding conventional CAR T cells targeting CD19, characterized by lower cytokine levels and broader range of antigens targeting. Therefore, this may offer a method of mitigating CRS, as well as a strategy for targeting other types of cancer, including solid tumors.

### 3.3. Other Strategies

In addition to the strategies mentioned above, it is possible to tune down the intrinsic potency of genetically targeted T cells by controlling the expression time or modulating the affinity of TCRs/CARs. The transient expression of CARs in T cells using nonviral methods (e.g., mRNA electroporation [12] and sleeping beauty transposition [94]) and the stimulation of activation-induced T-cell inhibitory proteins (e.g., PD-1 [95]) ensure the limited persistence of the redirected T cells; conversely, the regulation of affinity may be achieved via high-affinity TCR/CAR detection [96]. A fully human CAR comprised of the human C4 folate receptor-alpha (*α*FR)-specific scFv has been developed with lower affinity for *α*FR protein and less recognition of normal cells expressing low levels of *α*FR, which may overcome the issues of transgene immunogenicity and “on-target off-tumor” toxicity [97]. However, affinity tuning may decrease the threshold for CAR T-cell activation, which may change the therapeutic window of CAR T cells to tissues that express only high levels of antigen [98].

Besides, directing CAR T-cell delivery on the tumor sites anatomically may also limit toxicity and enhance therapeutic efficacy, which may be achieved by intratumoral or local intralymphatic delivery [99] and/or by engineering CAR T cells to express receptors of tumor-secreted chemokines [100, 101]. The “fourth-generation” CAR (or TRUCK) T cells with inducible release of IL-12 attract and activate innate immune cells to the targeted tumor lesion, which in turn eliminate cancer cells not recognized by CAR T cells [102, 103]. It offers a strategy to locally achieve therapeutic concentrations freed from systemic toxicity and prevent tumor relapse by residual cancer cells. Last but not least, the type of T cells used for adoptive transfer is critical, with T cells displaying a less differentiated phenotype potentially delivering improved therapy in vivo [104]. The CAR expressed in V*α*24-invariant natural killer T- (NKT-) cells can build on the natural antitumor properties of these cells while their restriction by monomorphic CD1d limits toxicity [105], and the CD19-transduced T memory stem cells cultured in IL-7 and IL-15 cytokines expanded more efficiently and showed more potent survival and more powerful antitumor effect in preclinical models [106].

## 4. Perspectives

Over the last decade, CAR-modified T-cell therapy has progressed rapidly, and dramatic benefits in patients with refractory hematological malignancies have formed a powerful trend in developing this therapy. The unparalleled efficacy was, however, frequently associated with toxicities that were not fully anticipated by preclinical studies. As better medical management of the associated adverse events has been put into effect and more innovative gene therapy strategies have been developed, we can expect that the era with improved control of toxicities with resulting superior outcomes and applicability of CAR T-cell approaches is not far away. The challenge will be to see whether in the next 5–10 years, the CAR T-cell approach will be more widely applied as the first-line treatment in a wider array of hematologic malignancies and other neoplasms.
